# Antioxidant and Starch-Hydrolyzing Enzymes Inhibitory Properties of *Striga*-Resistant Yellow-Orange Maize Hybrids

**DOI:** 10.3390/molecules26226874

**Published:** 2021-11-15

**Authors:** Abdulazeez Olamilekan Elemosho, Emmanuel Anyachukwu Irondi, Emmanuel Oladeji Alamu, Emmanuel Oladipo Ajani, Abebe Menkir, Busie Maziya-Dixon

**Affiliations:** 1 Department of Medical Biochemistry and Pharmacology, Kwara State University, Malete, PMB 1530, Ilorin 241103, Nigeria; elemoshoabdulazeezolamilekan@gmail.com (A.O.E.); emmanuel.irondi@kwasu.edu.ng (E.A.I.); emmanuel.ajani@kwasu.edu.ng (E.O.A.); 2 Food and Nutrition Sciences Laboratory, International Institute of Tropical Agriculture (IITA), PMB 5320, Oyo Road, Ibadan 200285, Nigeria; b.maziya-dixon@cgiar.org; 3 Food and Nutrition Sciences Laboratory, International Institute of Tropical Agriculture, Southern Africa Research and Administration Hub (SARAH) Campus, P.O. Box 310142, Chelstone, Lusaka 10101, Zambia; 4 Maize Breeding Unit, IITA, PMB 5320, Oyo Road, Ibadan 200285, Nigeria; a.menkir@cgiar.org

**Keywords:** total phenolics, total flavonoids, tannins, carotenoids, phytic acid, alpha-glucosidase inhibition assay, alpha-amylase inhibition assay

## Abstract

Most of the health benefits derived from cereals are attributed to their bioactive compounds. This study evaluated the levels of the bioactive compounds, and the antioxidant and starch-hydrolyzing enzymes inhibitory properties of six pipeline *Striga*-resistant yellow-orange maize hybrids (coded AS1828-1, 4, 6, 8, 9, 11) in vitro. The maize hybrids were grown at the International Institute of Tropical Agriculture (IITA), Nigeria. The bioactive compounds (total phenolics, tannins, flavonoids, and phytate) levels, antioxidant (DPPH^•^ and ABTS^•+^ scavenging capacity and reducing power) and starch-hydrolyzing enzymes (α-amylase and α-glucosidase) inhibitory activities of the maize hybrids were determined by spectrophotometry. At the same time, carotenoids were quantified using a reverse-phase HPLC system. The ranges of the bioactive compounds were: 11.25–14.14 mg GAE/g (total phenolics), 3.62–4.67 mg QE/g (total flavonoids), 3.63–6.29 mg/g (tannins), 3.66–4.31% (phytate), 8.92–12.11 µg/g (total xanthophylls), 2.42–2.89 µg/g (total β-carotene), and 3.17–3.77 µg/g (total provitamin A carotenoids). Extracts of the maize hybrids scavenged DPPH^•^ (SC_50_: 9.07–26.35 mg/mL) and ABTS^•+^ (2.65–7.68 TEAC mmol/g), reduced Fe^3+^ to Fe^2+^ (0.25 ± 0.64–0.43 ± 0.01 mg GAE/g), and inhibited α-amylase and α-glucosidase, with IC_50_ ranges of 26.28–52.55 mg/mL and 47.72–63.98 mg/mL, respectively. Among the six clones of the maize hybrids, AS1828-9 had the highest (*p* < 0.05) levels of tannins and phytate and the strongest antioxidant and starch-hydrolyzing enzymes inhibitory activities. Significant correlations were observed between total phenolics and the following: ABTS^•+^ (*p* < 0.01, r = 0.757), DPPH^•^ SC_50_ (*p* < 0.01, r = −0.867), reducing power (*p* < 0.05, r = 0.633), α-amylase IC_50_ (*p* < 0.01, r = −0.836) and α-glucosidase IC_50_ (*p* < 0.05, r = −0.582). Hence, the *Striga*-resistant yellow-orange maize hybrids (especially AS1828-9) may be beneficial for alleviating oxidative stress and postprandial hyperglycemia.

## 1. Introduction

The provision of high-yielding, nutritious and healthy food crops is essential in order to address the challenges posed by malnutrition and the diseases associated with it. Hence, different systematic approaches, such as breeding disease-resistant staples with improved nutritional quality, have been employed in plant breeding research programs to meet the dietary needs of the rapidly increasing global population. This has become more imperative considering the drastic reduction in the quantity and quality of global food supply occasioned by environmental pollution, global warming, and the development of novel biofuel sources [1]. In sub-Saharan Africa, maize (*Zea mays* L.) has been shown to contribute to food security and poverty reduction among low-income families [2]. 

Yellow-orange maize genotypes are well-known for their excellent nutritional and health-promoting qualities due to the bioactive compounds they contain, including carotenoids, polyphenolic compounds, phytic acid [3,4,5], and tocopherols [6]. However, the prospect of food security and improved nutrition through increased maize production in sub-Saharan Africa is threatened by the root hemi-parasitic plant, *Striga hermonthica* (Del.) Benth, which causes yield losses as high as 100% under severe infestation due to loss of water and nutrients through parasitism [7,8]. Consequently, *S. hermonthica* poses a biotic limitation to maize production in sub-Saharan Africa, emanating from a paradigm shift from the traditional system of farming cereals that involved extended fallow periods. Such a traditional system of farming cereals ensured that the level of *Striga* seed bank in the soil was tolerable to the plants [9]. To mitigate the losses in the yield and quality of maize occasioned by *S. hermonthica* infestation, the best control method is to plant *Striga*-resistant maize genotypes. This strategy is easily adaptable, especially in combination with other management practices [10].

Our recent study revealed that *Striga*-resistant yellow-orange maize hybrids contain carotenoids, polyphenols, and phytic acid [3]. These bioactive compounds are known to act as antioxidants and suppress postprandial hyperglycemia, among other health benefits [4,5,11,12]. Among the bioactive compounds in orange maize grain, the antioxidant [13,14] and digestive enzymes inhibitory activities [15] were reported to depend on the phenolic compounds, which exist mainly in the bound form. In addition, phenolic compounds are known to possess several other bioactivities such as inhibition of xanthine oxidase and angiotensin 1-converting enzymes, which are implicated in the pathogenesis of gout and rennin-dependent hypertension, respectively [11], and anti-inflammatory, anti-microbial, anti-cancer, anti-Alzheimer and anti-allergic properties among other biological activities [16]. Hence, phenolic compounds from dietary sources are attracting a great deal of attention from both scientists and consumers because of their benefits to human health [16]. 

The objective of this study was to evaluate the levels of the bioactive compounds, and the antioxidant and starch-hydrolyzing enzymes inhibitory properties of six pipeline *Striga*-resistant yellow-orange maize hybrids in vitro. The study also tested the associations among the bioactive constituents, and the antioxidant and starch-hydrolyzing enzymes inhibitory activities of the six pipeline *Striga*-resistant yellow-orange maize hybrids.

## 2. Results and Discussion

### 2.1. Bioactive Components in Six Pipeline Striga-Resistant Yellow-Orange Maize Hybrids 

The bioactive components determined in the six pipeline *Striga*-resistant yellow-orange maize hybrids (AS1828-1 (AS1), AS1828-4 (AS4), AS1828-6 (AS6), AS1828-8 (AS8), AS1828-9 (AS9), AS1828-11 (AS11)) in this study included total phenolics, flavonoids, tannins, carotenoids, and phytic acid. The levels of total phenolics, total flavonoids, tannins, and phytic acid are presented in Table 1. The total phenolics ranged from 11.25 to 14.14 mg GAE/g in AS4 and AS9, respectively; total flavonoids ranged from 3.62 to 4.67 mg QE/g in AS11 and AS6, respectively; tannin content ranged from 3.64 to 6.29 mg/g in AS1 and AS9, respectively; and phytic acid content ranged from 3.66% in AS6 to 4.47% in AS1.

Thus, AS9 contained the highest (*p* < 0.05) total phenolics and tannin levels, while AS6 had the highest total flavonoids content. The total phenolic concentrations detected in the *Striga*-resistant yellow-orange maize hybrids are higher than the values previously reported in yellow maize hybrids, including 2.15 mg GAE/g [15] and 2.08 mg GAE/g [17]. Similarly, the levels of total flavonoids detected in the *Striga*-resistant yellow-orange maize hybrids in this study are higher than the 0.93 ± 0.03 mg QE/g recently reported in provitamin A yellow maize flour [17]. Although the tannin levels are within the range (2.1–7.3 mg/g) previously reported in *Striga*-resistant yellow-orange maize hybrids [3], the values are comparatively higher than the range of condensed tannins (33.70 to 158.55 mg/100 g, equivalent to 0.34 to 1.59 mg/g) reported in pigmented maize genotypes [13].

The higher levels of total phenolics, total flavonoids, and tannins observed in the six pipeline *Striga*-resistant yellow-orange maize hybrids, relative to values in the existing literature for these polyphenolics in non-*Striga*-resistant yellow maize genotypes may be linked to possible differences in their genetic makeup [3]. It is well-known that the biosynthesis of polyphenolics and other plant secondary metabolites increases in the presence of stressors as a cellular defense and/or adaptive mechanism by the plant to withstand unfavorable conditions [16,18]. Moreover, *Striga* seed germination is stimulated by the production of strigolactones (plant hormones) in the roots of the maize plant, which the plant releases under stress [19]. Thus, it is possible that the trait for resistance to *S. hermonthica* may have up-regulated the biosynthesis of polyphenolic compounds in the maize to withstand the parasite. This is supported by an earlier report that post-attachment resistance to *S. hermonthica* involved thickening of the plant cell wall and the accumulation of many small vacuoles and phenolic deposits that are densely stained within the plant cell [20].

Phenolic compounds are notable for their antioxidant activity due to their redox properties, which enable them to act as quenchers of singlet oxygen, donors of hydrogen and reducing agents [21]. In addition, phenolic compounds were also reported to inactivate digestive enzymes, including pancreatic lipase, α-amylase, and α-glucosidase via non-specific binding to the individual enzymes [22]. As previously reported by Villiger et al. [23], phenolic compounds possess a high affinity for proteins via hydrogen and hydrophobic bonding, enhancing their ability to inhibit enzymes such as α-amylase and α-glucosidase by denaturation of protein. 

The phytic acid contents were comparable (*p* > 0.05) among the six pipeline *Striga*-resistant yellow-orange maize hybrids. This range agrees with our previous report on the phytic acid content of *Striga*-resistant yellow-orange maize hybrids [3]. Phytic acid possesses antioxidant activity, in addition to its inhibitory effect against the development of kidney stones [24], as well as anti-cancer properties [25]. The antioxidant properties of the bioactive components, especially the phenolic constituents (flavonoids and tannins) in the *Striga*-resistant yellow-orange maize hybrids, may also prevent and/or decelerate the oxidative degradation of some endogenous nutrients in the maize that are highly prone to oxidation, such as unsaturated fatty acids and vitamins [16]. Furthermore, the bioactive components in the *Striga*-resistant yellow-orange maize hybrids may reduce the rate at which some toxic oxidative products are formed, thus maintaining the nutritional quality and extending the shelf-life of food products [26] made from them.

The carotenoid content in the *Striga*-resistant yellow-orange maize hybrids are presented in Table 2. Total *β*-carotene (9-*cis*-*β*-carotene + 13-*cis*-*β*-carotene + all *trans*-*β*-carotene) ranged from 2.42 to 2.89 µg/g; total xanthophylls (lutein + zeaxanthin) ranged from 8.92 to 12.11 µg/g; and total provitamin A carotenoids (*β*-cryptoxanthin + *β*-carotene + *α*-carotene) ranged from 3.17 to 3.77 µg/g, in AS6 and AS9, respectively. There were no significant differences (*p* > 0.05) in the carotenoid content of the *Striga*-resistant yellow-orange maize hybrids. The ranges of carotenoids obtained in this study confirm those previously reported by Alamu et al. for provitamin A biofortified yellow maize hybrids [27]. Furthermore, the total xanthophylls were higher in value than the total provitamin A carotenoids in the *Striga*-resistant yellow-orange maize hybrids, corroborating the findings of Ortiz et al. [28]. 

The carotenoids may have complemented the antioxidant and starch-hydrolyzing enzymes inhibitory activities of the phenolic compounds in the *Striga*-resistant yellow-orange maize hybrids, in accordance with their reported bioactivities. For example, carotenoids have been reported to possess antioxidant activity as the main mechanism underlying their health benefits [29]. They also confer protective effects against non-communicable chronic diseases such as cancer [30] and cardiovascular diseases [31]. Also, *β*-cryptoxanthin was reported to significantly reduce the risk of type 2 diabetes (T2D) and mitigate insulin resistance [32,33].

### 2.2. Antioxidant Activity of Six Pipeline Striga-Resistant Yellow-Orange Maize Hybrids 

The antioxidant activity of the *Striga*-resistant yellow-orange maize hybrids (Table 3) revealed that the six pipeline clones all exhibited antioxidant activity by scavenging free radicals (ABTS^•^+ and DPPH^•^) and reducing ferric ions (Fe^3+^) to ferrous ions (Fe^2+^). The antioxidant activity varied significantly (*p* < 0.05) among the hybrids, with AS9 having the strongest (*p* < 0.05) free radicals scavenging abilities (7.28 TEAC mmol/g and SC_50_, 9.07 ± 0.27 mg/mL for ABTS^•+^ and DPPH^•^, respectively) and ferric-reducing power (0.43 mg GAE/g). It is pertinent to recall that AS9-9 also had the highest level of total phenolics and tannins, as presented earlier in Table 1. The DPPH^•^ scavenging abilities of the *Striga*-resistant yellow-orange maize hybrids obtained in this study (SC_50_: 9.07 to 26.35 mg/mL) are more potent than those reported by Rodríguez-Salinas et al. [13] for pigmented maize genotypes (IC_50_: 31 to 52 mg/mL) since a lower SC_50_ or IC_50_ is indicative of a stronger activity [34]. However, vitamin C, a standard antioxidant with a lower SC_50_ (4.63 ± 0.28 mg/mL), had a stronger DPPH^•^ scavenging activity than all of the six *Striga*-resistant yellow-orange maize hybrids. Similarly, the ABTS^•+^ scavenging activity of the *Striga*-resistant yellow-orange maize hybrids (2.65–7.68 TEAC mmol/g) is higher than the value (294.81 ± 2.23 μmol TEAC/g) reported by Irondi et al. [17] for provitamin A yellow maize flour. The stronger antioxidant activity of the *Striga*-resistant yellow-orange maize hybrids over the non-*Striga*-resistant pigmented maize genotypes may be attributed to the increased deposition of polyphenolic compounds in their defense against *S. hermonthica* [20], which may have, consequently, enhanced the antioxidant capacity of the *Striga*-resistant yellow-orange maize hybrids.

The free radicals scavenging ability and ferric-reducing power of the *Striga*-resistant yellow-orange maize hybrids suggest that they may be beneficial in protecting the body from the oxidative assaults precipitated by free radicals and reactive oxygen species. Thus, the *Striga*-resistant yellow-orange maize hybrids may have a protective effect against the oxidative damage of biomolecules in the body, including nucleic acids, proteins, lipids and carbohydrates [35] and the chronic diseases related to oxidative stress [36].

### 2.3. Starch-Hydrolyzing Enzymes Inhibitory Activities of the Six Pipeline Striga-Resistant Biofortified Yellow-Orange Maize Hybrids

The starch-hydrolyzing enzymes (α-amylase and α-glucosidase) inhibitory activity of the six pipeline *Striga*-resistant yellow-orange maize hybrids, expressed as IC_50_ (extract concentration that inhibited enzyme activity by 50%), is presented in Table 4. The IC_50_ values of the *Striga*-resistant yellow-orange maize hybrids on α-amylase and α-glucosidase ranged from 26.28 to 52.55 mg/mL and 47.72 to 63.98 mg/mL in AS9 and AS4, respectively. Thus, among the six pipeline *Striga*-resistant yellow-orange maize hybrids, AS9 with the lowest IC_50_ values for both α-amylase and α-glucosidase, displayed the strongest (*p* < 0.05) inhibitory activity on these two enzymes. Interestingly, there was no significant (*p* > 0.05) difference in the IC_50_ values of AS9 and acarbose (a standard antidiabetic drug) on α-amylase, indicating that the α-amylase inhibitory abilities of AS9 and acarbose were comparable. However, except for the α-amylase inhibitory ability of AS9 that was comparable with that of acarbose, the α-amylase and α-glucosidase inhibitory activities of the acarbose were stronger than those of the *Striga*-resistant yellow-orange maize hybrids. The ability of different pigmented (yellow, purple, red, and black) maize genotypes to inhibit starch-hydrolyzing enzyme (α-glucosidase) activity was reported by Fabila-Garca et al. [15]. Their findings revealed that yellow corn extract had the highest α-glucosidase inhibitory activity, expressed as a percentage (69.8%), among the maize genotypes. In addition, Irondi et al. [17] recently reported α-amylase and α-glucosidase IC_50_ values of 237.12 ± 2.60 and 157.18 ± 1.05 μg/mL, respectively, for provitamin A yellow maize flour. Relative to corn silk extract, which was reported [37] to inhibit α-amylase and α-glucosidase with average IC_50_ values of 218.4 and 221.4 µg/mL, respectively, the six pipeline *Striga*-resistant yellow-orange maize hybrids had a weaker inhibitory effect on α-amylase and α-glucosidase. 

Both α-amylase and α-glucosidase are involved in the digestion of dietary carbohydrates. Whereas α-amylase in the small intestine hydrolyzes starch α-1,4 bonds to release oligosaccharides and disaccharides, α-glucosidase in the brush border of the small intestine completes the digestion by further hydrolyzing the oligosaccharides and disaccharides to yield absorbable monosaccharides, including glucose and fructose [38]. Hence, the inhibition of these two digestive enzymes is a well-established therapeutic approach for alleviating postprandial hyperglycemia in T2D management and a key mechanism of action of many anti-diabetic agents [39], including drugs, natural products and functional foods. Furthermore, the *Striga*-resistant yellow-orange maize hybrids had a more substantial inhibitory effect on α-amylase than on α-glucosidase. This pattern of starch-hydrolyzing enzymes inhibition has beneficial therapeutic implications and agrees with the pattern reported in previous studies [17,40]. Thus, the six pipeline *Striga*-resistant yellow-orange maize hybrids, especially AS9, may have some benefits in controlling postprandial hyperglycemia.

### 2.4. Correlations between the Bioactive Components, Antioxidant and Starch-Hydrolyzing Enzymes Inhibitory Activities of the Six Pipeline Striga-Resistant Yellow-Orange Maize Hybrids

Among the bioactive components, total phenolics significantly correlated with ABTS^•^+ (*p* < 0.01, r = 0.757), DPPH^•^ SC_50_ (*p* < 0.01, r = -0.867), reducing power (*p* < 0.05, r = 0.633), α-amylase IC_50_ (*p* < 0.01, r = −0.836) and α-glucosidase IC_50_ (*p* < 0.05, r = −0.582) (Table 5). As earlier stated, lower DPPH^•^ SC_50_ and enzyme IC_50_ values are indicative of stronger scavenging and inhibitory activities of a given sample on DPPH^•^ and enzymes, respectively [34]. Thus, when taken together, the negative correlations between total phenolics and DPPH^•^ SC_50_, α-amylase IC_50_ and α-glucosidase IC_50_, as well as the positive correlations between total phenolics and ABTS^•+^ scavenging ability and reducing power, suggest that phenolic compounds may have contributed majorly to the observed antioxidant and starch-hydrolyzing enzymes inhibitory activities of the *Striga*-resistant yellow-orange maize hybrids. 

## 3. Materials and Methods

### 3.1. Chemicals and Reagents 

Trolox, quercetin, L-ascorbic acid, gallic acid, ABTS (2,2′-azino-bis-(3-ethylbenzthiazoline-6-sulphonic acid)), DPPH (2,2-diphenyl-2-picrylhydrazyl), α-glucosidase from *Bacillus stearothermophillus*, *p*-nitrophenylglucopyranoside (PNPG), α-amylase, soluble starch and acarbose were purchased from Sigma (St. Louis). Analytical grades of all other chemicals and solvents were used.

### 3.2. Sample Collection 

Dry seed samples of six pipeline *Striga*-resistant yellow-orange maize hybrids (coded AS1828-1, 4, 6, 8, 9, 11) all grown at Saminaka (8°39′ E, 10°34′ N; altitude of 760 m; annual rainfall of 1149 mm; temperature of 18.1–37.3 °C; soil type, Dystric Nitosols) and Zaria (7°45′ E, 11°8′ N; altitude of 622 m; annual rainfall of 1076 mm; average temperature of 13.9–35.5 °C; soil type, Ferric Luvisols) were collected from the Maize Improvement Program of the International Institute of Tropical Agriculture (IITA), Ibadan, Nigeria. The hybrids were planted in May for two seasons, in a randomized complete block design in three replications, during the rainy season. The samples were milled into flour (0.50 mm sieve size) with Perten Laboratory Hammer Mill (3102, USA) and packed hermetically in opaque sample bags for further laboratory analyses.

### 3.3. Preparation of Samples’ Extract 

To prepare an extract from the *Striga*-resistant yellow-orange maize hybrids flour, 1 g of the flour was soaked in 10 mL of methanol in a covered 50 mL centrifuge tube overnight (12 h) with intermittent shaking. Subsequently, the mixture was centrifuged at 3000 rpm for 10 min, and then the supernatant (methanolic extract) was collected and stored at −4 °C until analysis [41].

### 3.4. Determination of Total Phenolics Content 

The Folin–Ciocalteu method described by Singleton et al. [42] was adopted to determine the total phenolics content of the *Striga*-resistant yellow-orange maize hybrids’ flour extract. A portion (300 μL) of the extract was dispensed into a test tube (in triplicates). Afterward, 1.5 mL of Folin–Ciocalteu reagent (stock Folin–Ciocalteu reagent diluted 10 times with distilled water) and 1.2 mL of Na_2_CO_3_ solution (7.5% *w*/*v*) were added, and the mixture was incubated in the dark for 30 min at room temperature. After that, the absorbance was read at 765 nm against a blank. The total phenolic content was calculated using a gallic acid calibration curve, and expressed as gallic acid equivalent (GAE) in mg/g sample. 

### 3.5. Determination of Total Flavonoids Content 

The protocol described by Kale et al. [43] was employed to determine the total flavonoids content of the *Striga*-resistant yellow-orange maize hybrids’ flour extract. Briefly, 0.5 mL of the extract was dispensed into test tubes; this was followed by adding 1.5 mL of methanol, 0.1 mL of aluminum chloride (10%), 0.1 mL of 1 M potassium acetate, and 2.8 mL of distilled water. The reaction mixture was vortexed and incubated at room temperature for 30 min, after which the absorbance was read at 514 nm. The total flavonoids content of the extracts was expressed as quercetin equivalent (QE) in mg/g sample.

### 3.6. Determination of Tannin Content

The tannin content of the *Striga*-resistant yellow-orange maize hybrids’ flour extracts was quantified by the colorimetric method previously described by Joslyn [44], with a slight modification. A portion of the sample (0.5 g) was dispersed in 5 mL of 1% HCl in methanol and left for 15 min. Afterward, the mixture was centrifuged at 3000 rpm for 10 min. A portion of 0.1 mL of the supernatant was dispensed into the test tube containing 7.5 mL of distilled water, following which 0.5 mL of Folin-Dennis reagent and 1 mL of Na_2_CO_3_ (35%) solution were added, and the volume was made up to 10 mL with 0.9 mL of distilled water. After mixing, the reaction mixture was incubated for 30 min at room temperature, and the absorbance was read at 760 nm. The tannin content, expressed as tannic acid equivalent (TAE) in mg/g sample, was calculated from a tannic acid standard curve.

### 3.7. Quantification of Carotenoid Content of the Sample

The carotenoid content of the *Striga*-resistant yellow-orange maize hybrids’ flour was determined by adopting the method described by Howe and Tanumihardjo [45]. Carotenoids were extracted from the flours by mixing 0.6 g of the sample with 6 mL of ethanol (containing 0.1% butylated hydroxyl toluene). The mixture was placed in a water bath at 85 °C for 5 min. Next, the interfering oil in the mixture was saponified with potassium hydroxide (80 % *w*/*v*) at 85 °C in a water bath for 5 min. The suspension was then mixed using a vortex machine and returned to the water bath for another 5 min. It was immediately transferred into a bath of ice, and 3 mL of cold deionized water was added. The carotenoid contents from the mixture were separated three consecutive times with 3 mL of n-hexane by centrifuging at 1000 rpm for 10 s. The upper layer of the mixture was dispensed into a 50 mL concentrator tube. The combined hexane fraction was washed thrice with deionized water, vortexed, and centrifuged for 10 s at 1000 rpm. The n-hexane fraction was dried down using a TurboVap (LIV) Concentrator under nitrogen gas for 25 min. The dried extract was reconstituted with methanol/dichloromethane (1 mL, 50:50 *v*/*v*), and a 100 µL aliquot was injected into the HPLC system to quantify the carotenoids. The HPLC system (Water Corporation, Milford, MA, USA) comprised a guard column, C30 YMC carotenoid column (4.6 × 250 mm, 3 µM), binary HPLC pump (Waters 626), auto-sampler (Waters 717), and a photodiode array detector (Waters 2996). The system operated with Empower 1 software (Waters Corporation). The mobile phase consisted of solvent A, containing methanol:water (92:8 *v*/*v*) with 10 mmol/L ammonium acetate, and solvent B, containing 100% methyl tertiary-butyl ether. Gradient elution was performed at a flow rate of 1 mL/min under the following conditions: 29 min of linear gradient from 83% to 59% A; 6 min of linear gradient from 59% to 30% A; 1 min of hold at 30% A; 4 min of linear gradient from 30% to 83% A and a 4-min hold at 83%. Chromatograms of the carotenoids were generated at 450 nm, and the specific carotenoids were identified and quantified using the external standards method based on the calibration curve from pure standards and comparison of the absorption spectrum and co-elution with standard carotenoids.

### 3.8. Determination of Phytic Acid Content

The method of Wheeler and Ferrel [46] was employed to determine the phytic acid content of the flours. Extraction was done by mechanically shaking a mixture of 1 g of flour and 25 mL of 3% trichloroacetic acid (TCA) for 1 h and the suspension was centrifuged for 15 min at 3500 rpm. A 10 mL aliquot of the supernatant was mixed with a 4 mL ferric chloride solution, and the mixture was heated in a boiling water bath for 45 min. The resulting suspension was centrifuged at 3500 rpm for 15 min and the supernatant was carefully decanted. Thereafter, the precipitate was washed twice by dispersing in 25 mL of 3% TCA, heating in a boiling water bath for 10 min, and centrifuging at 3500 rpm for 10 min. The precipitate volume was made up to 30 mL with distilled water, and the mixture was heated in a boiling water bath for 30 min. The hot suspension was filtered with the aid of Whatman filter paper (No 2), and the precipitate was washed with 60 mL of hot distilled water to ensure complete filtration. Next, the precipitate retained on the filter paper was dissolved with 40 mL of hot 3.2 M HNO_3_ into a 100 mL volumetric flask. A 0.5 mL aliquot was transferred into a centrifuge tube and diluted with 7 mL of distilled water, after which 2 mL of 1.5 M KSCN was added, and the volume was made to 10 mL with 0.5 mL distilled water. Absorbance was read (within 1 min) at 480 nm. The phytic acid content of the flours was calculated using a Fe/P atomic ratio of 4:6.

### 3.9. 2,2-Azinobis(3-ethyl-benzothiazoline-6-sulfonic Acid) Radical Cation (ABTS^•+^) Scavenging Assay 

The ability of the *Striga*-resistant yellow-orange maize hybrids’ flour extracts to scavenge ABTS^•+^ was investigated by adopting the procedure reported by Re et al. [47]. ABTS^•+^ working reagent was prepared by thoroughly mixing equal volume of aqueous solutions of ABTS^•+^ (7 millimole/L) and K_2_S_2_O_8_ (2.45 millimole/L) and incubating the mixture in a dark cupboard at room temperature for 16 h. Afterward, the reagent’s absorbance was adjusted to 0.70 ± 0.02 with ethanol (95%) at 734 nm. Then 0.2 mL of the extract and 2.0 mL of the ABTS^•+^ reagent were dispensed into the test tube, mixed well, and incubated at room temperature for 15 min in a dark condition. Finally, the absorbance was read in a UV-Visible spectrophotometer (Milton Roy Company, USA) at 734 nm. The ABTS•+ scavenging ability of the flour extracts was later calculated from a Trolox standard curve and was expressed as the Trolox equivalent antioxidant capacity (TEAC). 

### 3.10. 2,2-Diphenyl-2-picrylhydrazyl Radical (DPPH^•^) Scavenging Assay 

The protocol reported by Cervato et al. [48] was employed to determine the flour extracts’ ability to scavenge DPPH^•^, using vitamin C (ascorbic acid) as a reference antioxidant. Briefly, a reaction mixture containing 1.0 mL of different concentrations (8, 16, 24, 32 mg/mL) of the extract (or vitamin C) and 3.0 mL of DPPH^•^ solution (60 μM) was incubated at room temperature in a dark condition for 30 min. Thereafter, the absorbance was read at 517 nm, and the DPPH^•^ scavenging ability (%) of the extracts was calculated and expressed as the concentration of extract that scavenged 50% of DPPH^•^ (SC_50_).

$$\%\;\mathrm{scavenging ability}\;=\frac{(\mathrm{Abs}517_{\mathrm{ref}}\;-\;\mathrm{Abs}517_{\mathrm{sample}})\;\times \;100}{\mathrm{Abs}517_{\mathrm{ref}}}$$

where Abs517_ref_ is the absorbance reading of the reference test, and Abs517_sample_ is the absorbance reading of the sample’s extract.

### 3.11. Reducing Power Assay

The ability of the flour extracts to reduce Fe^3+^ to Fe^2+^ was tested by adopting the protocol described by Oyaizu [49]. In brief, a mixture of the extract (2.5 mL), 200 mM sodium phosphate buffer (pH 6.6) (2.5 mL), and 1% potassium ferricyanide (2.5 mL) was incubated at 50 °C for 20 min, after which 2.5 mL of 10% trichloroacetic acid was added. Next, the mixture was centrifuged at 650× *g* for 10 min. A portion of 2.5 mL of the supernatant was dispensed in a test tube, and 2.5 mL of distilled water and 1 mL of 0.1% ferric chloride were added and mixed thoroughly, and the absorbance was read at 700 nm. Finally, the reducing power of the extracts was calculated and expressed in milligram gallic acid equivalent per gram of sample.

### 3.12. Alpha-Amylase Inhibition Assay 

Alpha-amylase inhibition assay was conducted by adopting the procedure described by Kwon et al. [50]. Porcine pancreas α-amylase (EC 3.2.1.1) and soluble starch (substrate) were used in this assay. Different dilutions of the flour’s extracts, totaling 500 μL, and 500 μL of 0.02 M sodium phosphate buffer (pH 6.9 with 0.006 M NaCl) containing 0.5 mg/mL α-amylase solution were mixed and incubated at 37 °C for 10 min. Thereafter, 500 μL of 1% starch solution in 0.02 M sodium phosphate buffer was added, and the reaction mixture was incubated at 37 °C for 15 min. Subsequently, the α-amylase-catalyzed hydrolysis of starch was terminated by adding 1.0 mL of DNSA color reagent (1% 3,5-dinitrosalicylic acid, and 12% sodium potassium tartrate in 0.4 M NaOH). The reaction mixture was later incubated for 5 min in a boiling water bath, cooled to room temperature, and diluted with 10 mL distilled water. The absorbance of the mixture was read at 540 nm. A reference test that excluded the flours extract was included in the experiment. After that, the percentage α-amylase inhibition was calculated as follows:

$$\%\;\mathrm{Inhibition}\;=\frac{(A540R\;-\;A540S)\;\times \;100}{A540R}$$

where A540R is the absorbance reading of the reference; A540S is the absorbance reading of the sample.

### 3.13. Alpha-Glucosidase Inhibition Assay

Alpha-glucosidase inhibitory activity of the flours extracts was conducted by adopting the procedure reported by Kim et al. [39], using *Bacillus stearothermophillus* α-glucosidase (EC 3.2.1.20) and *para*-nitrophenylglucopyranoside (PNPG) as the substrate. Five (5) units of aliquot of α-glucosidase was incubated with 20 μg/mL of the extract for 15 min. The hydrolytic reaction was initiated by adding 3 mM PNPG prepared in 20 mM phosphate buffer, pH 6.9, which served as a substrate. The hydrolytic reaction was allowed to proceed for 20 min at 37 °C, after which 2 mL of 0.1 M Na_2_CO_3_ was added to terminate the reaction. A reference test without the flour extract was included in the experiment. The absorbance of the yellow *p*-nitrophenol released from the α-glucosidase-catalyzed hydrolysis of PNPG was read at 400 nm and the percentage α-glucosidase inhibition was calculated thus:

$$\%\;\mathrm{Inhibition}\;=\frac{(A400R\;-\;A400S)\;\times \;100}{A400R}$$

where A400R is the absorbance reading of the reference; A400S is the absorbance reading of the sample.

### 3.14. Data Analysis

The data obtained in this study (from triplicate determinations) were expressed as mean values ± standard deviation (SD). Using the SPSS statistical software package (16th version), one-way analysis of variance (ANOVA) was performed on the data, and the mean values were compared using Tukey’s post hoc test at *p* ˂ 0.05. The associations between the bioactive components, the antioxidant and the starch-hydrolyzing enzymes inhibitory activities were calculated using the Pearson correlation test. Column representations of the mean values were done using GraphPad prism (5th version).

## 4. Conclusions

The six pipeline *Striga*-resistant yellow-orange maize hybrids contained important bioactive constituents (total phenolics, total flavonoids, tannins, phytic acid and carotenoids). Their extracts exhibited strong antioxidant activity and inhibited starch-hydrolyzing enzymes (α-amylase and α-glucosidase). Among the *Striga*-resistant yellow-orange maize hybrids, AS1828-9 had the most potent antioxidant and starch-hydrolyzing enzyme inhibitory activities. Significant correlations were observed between total phenolic content and the ABTS^•+^, DPPH^•^ scavenging ability, reducing power, α-amylase, and α-glucosidase inhibitory activity of the *Striga*-resistant yellow-orange maize hybrids. The antioxidant and starch-hydrolyzing enzymes inhibitory activities suggest that the *Striga*-resistant yellow-orange maize hybrids (especially AS1828-9) may be beneficial in preventing and/or alleviating oxidative stress and postprandial hyperglycemia.

## Figures and Tables

**Table 1:** Total phenolics, flavonoids, tannins, and phytate content of six pipeline *Striga*-resistant yellow-orange maize hybrids.

Hybrid	Total Phenolics (mg GAE/g)	Flavonoids (mg QE/g)	Tannins (mg/g)	Phytate (%)
AS1828-1	13.25 ± 0.16 ^b^	4.59 ± 0.11 ^a^	3.64 ± 0.02 ^a^	4.47 ± 0.50 ^a^
ASI828-4	11.25 ± 0.25 ^a^	4.31 ± 0.13 ^a,b^	4.67 ± 0.01 ^b^	4.07 ± 0.40 ^a^
AS1828-6	14.07 ± 0.06 ^c^	4.67 ± 0.40 ^a^	4.29 ± 0.42 ^b^	3.66 ± 0.85 ^a^
AS1828-8	12.66 ± 0.13 ^b^	4.24 ± 0.09 ^a,b^	5.33 ± 0.16 ^c^	3.77 ± 0.50 ^a^
AS1828-9	14.14 ± 0.12 ^c^	4.17 ± 0.13 ^a,b^	6.29 ± 0.42 ^d^	4.18 ± 0.48 ^a^
AS1828-11	13.06 ± 0.28 ^b^	3.62 ± 0.22 ^a^	5.34 ± 0.16 ^c^	4.28 ± 1.72 ^a^

Results are mean values ± standard deviation (SD) of independent triplicate determinations. Along the same column, values having the same superscript letter do not vary significantly (*p* > 0.05).

**Table 2:** Carotenoid content of six pipeline *Striga*-resistant yellow-orange maize hybrids.

Hybrid	Total *β*-Carotene (µg/g)	Total Xanthophylls (µg/g)	Total provitamin A Carotenoids (µg/g)
AS1828-1	2.54 ± 0.80 ^a^	10.96 ± 4.17 ^a^	3.72 ± 1.35 ^a^
AS1828-4	2.59 ± 0.60 ^a^	12.10 ± 1.33 ^a^	3.40 ± 0.81 ^a^
AS1828-6	2.42 ± 0.88 ^a^	8.92 ± 2.90 ^a^	3.17 ± 1.18 ^a^
AS1828-8	2.46 ± 0.31 ^a^	10.36 ± 1.15 ^a^	3.20 ± 0.41 ^a^
AS1828-9	2.89 ± 0.94 ^a^	12.11 ± 2.67 ^a^	3.77 ± 1.24 ^a^
AS1828-11	2.79 ± 0.63 ^a^	11.50 ± 2.66 ^a^	3.72 ± 1.32 ^a^

Results are mean values ± standard deviation (SD) of independent triplicate determinations. Along the same column, values having the same superscript letter do not vary significantly (*p* > 0.05).

**Table 3:** DPPH^•^ SC_50_, ABTS^•+^ scavenging ability and reducing power of six pipeline *Striga*-resistant yellow-orange maize hybrids.

Hybrid	DPPH^•^ SC_50_ (mg/mL)	ABTS^•+^ Scavenging Ability (mmol TEAC/g)	Reducing Power (mg GAE/g)
AS1828-1	16.85 ± 0.50 ^c^	4.00 ± 0.50 ^a^	0.30 ± 0.06 ^a^
AS1828-4	26.35 ± 0.30 ^d^	2.65 ± 0.21 ^a^	0.25 ± 0.64 ^b^
AS1828-6	12.58 ± 0.17 ^b^	5.28 ± 0.21 ^b^	0.36 ± 0.23 ^b^
AS1828-8	12.95 ± 0.64 ^b^	4.41 ± 0.33 ^b^	0.43 ± 0.01 ^c^
AS1828-9	9.07 ± 0.27 ^a^	7.68 ± 1.50 ^d^	0.42 ± 0.02 ^c^
AS1828-11	12.56 ± 1.24 ^b^	6.08 ± 0.28 ^c^	0.39 ± 0.02 ^b^
Ascorbic acid	4.63 ± 0.28	-	-

Results are mean values ± standard deviation (SD) of independent triplicate determinations. Along the same column, values having different superscript letters vary significantly (*p* < 0.05). SC_50_, concentration of extract that scavenged 50% of DPPH^•^; TEAC, trolox equivalent antioxidant capacity; GAE, gallic acid equivalent.

**Table 4:** Alpha-amylase and α-glucosidase IC_50_ of six pipeline *Striga*-resistant yellow-orange maize hybrids.

Hybrid	α-Amylase IC_50_ (mg/mL)	α-Glucosidase IC_50_ (mg/mL)
AS1828-1	39.90 ± 0.31 ^d^	61.97 ± 2.5 ^e^
AS1828-4	52.55 ± 0.64 ^e^	63.98 ± 0.68 ^e^
AS1828-6	35.66 ± 0.20 ^c^	58.05 ± 1.77 ^d^
AS1828-8	36.54 ± 0.42 ^c^	55.16 ± 0.37 ^d^
AS1828-9	26.28 ± 0.35 ^a^	47.72 ± 0.40 ^b^
AS1828-11	30.10 ± 1.27 ^b^	51.83 ± 2.79 ^c^
Acarbose	24.45 ± 0.06 ^a^	32.88 ± 2.65 ^a^

Results are mean values ± standard deviation (SD) of independent triplicate determinations. Along the same column, values having different superscript letters vary significantly (*p* < 0.05). IC_50_, concentration of extract that inhibited 50% of enzyme activity.

**Table 5:** Correlations between the bioactive components, antioxidant and starch-hydrolyzing enzymes inhibitory activities of six pipeline *Striga*-resistant yellow-orange maize hybrids.

Parameter	ABTS^•+^ Scavenging Ability (mmol TEAC/g)	DPPH^•^ SC_50_ (mg/mL)	Reducing Power (mg GAE/g)	α-Amylase IC_50_ (mg/mL)	α–Glucosidase IC_50_ (mg/mL)
Total phenolics (mg GAE/g)	0.757 **	−0.867 **	0.633 *	−0.836 **	− 0.582 *
Tannins (mg TAE/g)	0.641 *	−0.464	0.689 *	− 0.555	−0.831 **
Total flavonoids (mg QE/g)	−0.291	0.195	−0.193	0.362	0.460
Phytate (%)	0.589 *	−0.334	0.195	−0.430	−0.314

*, Correlation is significant at *p* ≤ 0.05 level; **, correlation is significant at *p* ≤ 0.01 level; GAE, gallic acid equivalent; TAE, tannic acid equivalent; QE, quercetin equivalent; TEAC, trolox equivalent antioxidant capacity; SC_50_, concentration of extract that scavenged 50% of DPPH^•^; IC_50_, concentration of extract that inhibited 50% of enzyme activity.

## Data Availability

The data supporting the results reported in this paper are available on request from the corresponding author (e-mail: o.alamu@cgiar.org).

## Sample Availability

Samples of the six pipeline *Striga*-resistant yellow-orange maize hybrids are available on request from the authors.
